# Supranormal orientation selectivity of visual neurons in orientation-restricted animals

**DOI:** 10.1038/srep16712

**Published:** 2015-11-16

**Authors:** Kota S. Sasaki, Rui Kimura, Taihei Ninomiya, Yuka Tabuchi, Hiroki Tanaka, Masayuki Fukui, Yusuke C. Asada, Toshiya Arai, Mikio Inagaki, Takayuki Nakazono, Mika Baba, Daisuke Kato, Shinji Nishimoto, Takahisa M. Sanada, Toshiki Tani, Kazuyuki Imamura, Shigeru Tanaka, Izumi Ohzawa

**Affiliations:** 1 Graduate School of Frontier Biosciences, Osaka University, Suita, Osaka 565-0871, Japan; 2 Center for Information and Neural Networks (CiNet), National Institute of Information and Communications Technology (NICT), Suita, Osaka 565-0871, Japan; 3 Universität Tübingen, 72076 Tübingen, Germany; 4 Primate Research Institute, Kyoto University, Inuyama, 484-8506, Japan; 5 Kyoto Sangyo University, Kita-ku, Kyoto 603-8555, Japan; 6 National Institute for Physiological Sciences, Okazaki, Aichi 444-8585, Japan; 7 RIKEN Brain Science Institute, Wako, Saitama 351-0198, Japan; 8 Graduate School of Medicine, Hirosaki University, Aomori 036-8562, Japan; 9 Department of Systems Life Engineering, Maebashi Institute of Technology, Gunma 371-0816, Japan; 10 Brain Science Inspired Life Support Research Center, The University of Electro-Communications, Tokyo 182-8585, Japan

## Abstract

Altered sensory experience in early life often leads to remarkable adaptations so that humans and animals can make the best use of the available information in a particular environment. By restricting visual input to a limited range of orientations in young animals, this investigation shows that stimulus selectivity, e.g., the sharpness of tuning of single neurons in the primary visual cortex, is modified to match a particular environment. Specifically, neurons tuned to an experienced orientation in orientation-restricted animals show sharper orientation tuning than neurons in normal animals, whereas the opposite was true for neurons tuned to non-experienced orientations. This sharpened tuning appears to be due to elongated receptive fields. Our results demonstrate that restricted sensory experiences can sculpt the supranormal functions of single neurons tailored for a particular environment. The above findings, in addition to the minimal population response to orientations close to the experienced one, agree with the predictions of a sparse coding hypothesis in which information is represented efficiently by a small number of activated neurons. This suggests that early brain areas adopt an efficient strategy for coding information even when animals are raised in a severely limited visual environment where sensory inputs have an unnatural statistical structure.

The perceptual and cognitive abilities of humans and animals often develop by adapting to a particular environment^1^. Early life experience is especially critical for these abilities by influencing the functional development of neurons in the early sensory areas of the brain^2^,^3^,^4^,^5^,^6^,^7^,^8^,^9^,^10^,^11^,^12^. One of the most well-known examples of altered sensory experience in early life is orientation-restricted rearing, which causes a loss of neurons that are responsive to the deprived orientation in the primary visual cortex^2^,^3^,^4^,^5^,^6^,^7^,^8^,^9^. This results in an alteration in the distribution of the preferred orientation of neurons: neurons tuned to the experienced orientation dominate the primary visual cortex. Therefore, sensory inputs are represented in a highly redundant manner in orientation-restricted animals with neurons signalling the same orientation.

How does the primary visual cortex deal with such redundancy? One potential solution to this problem is sharpening the orientation tuning of individual neurons; if the orientation tuning curves of single neurons are sharpened to cover a very limited range, such an adaptation allows different sets of neurons to be activated for different orientation stimuli even in a restricted orientation range. In fact, a computational study suggested that they should^13^, but no physiological study has examined this issue. Do single neurons in primary sensory areas show any functional properties tailored to a particular environment as a consequence of adaptation? To answer this question, we investigated whether neurons signalling an experienced orientation exhibit differences from those signalling unfamiliar orientations in area 17 of orientation-restricted animals, and compared the results with neurons from normal animals.

## Results

### Orientation is restricted with chronically mounted goggles

**Figure 1:**
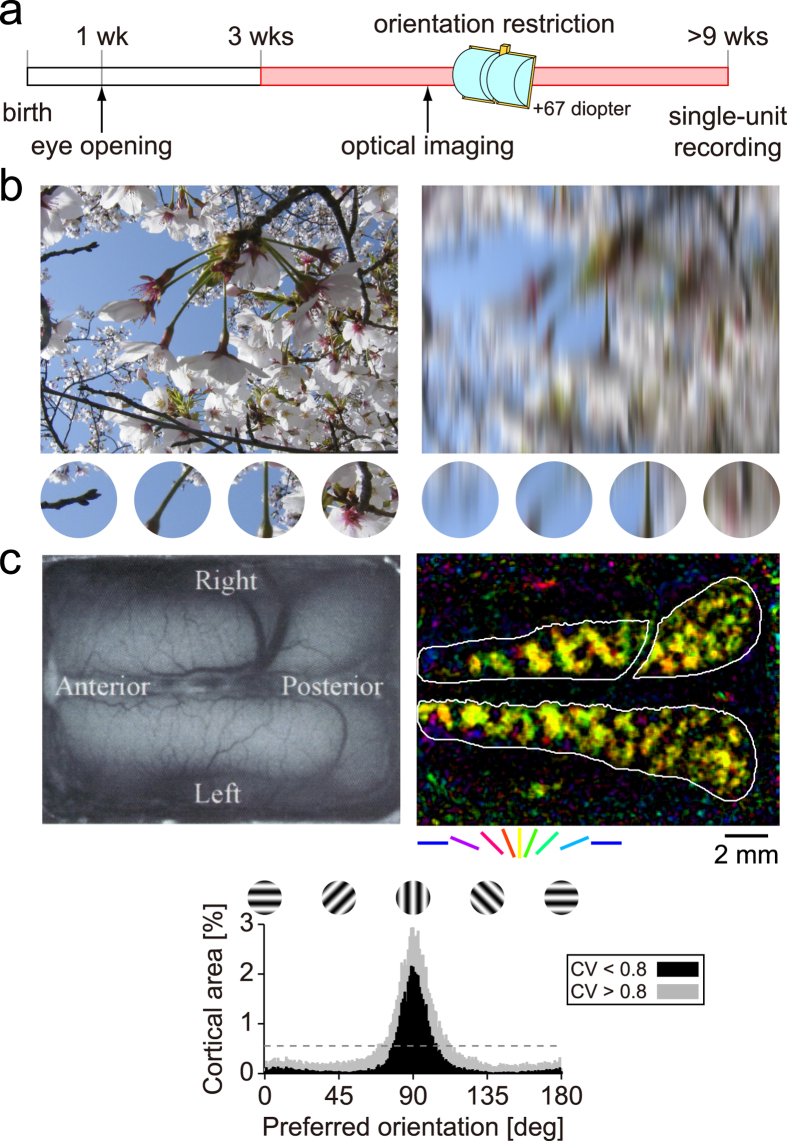
Orientation restriction with v-goggles. (**a**) Typical experiment schedule. (**b**) Simulated defocus by cylindrical lenses. Left, Original natural image. Small image patches are extracted to show local orientation structures (magnified ×1.75). Right, Defocused image (+67 diopter). Note the disappearance of horizontal and oblique features. The photograph was taken by K. S. Sasaki. (**c**) Top left, Cortical image of area 17/18. Top right, Orientation map by intrinsic optical imaging at 36 days after birth. Colour and brightness indicate the preferred orientation and signal magnitude, respectively. The average of responses at 0.15 and 0.5 cpd is shown. The white border delineates the extent of area 17 where 0.5 cpd gratings elicited higher responses than 0.15 cpd gratings. Bottom, Distribution of preferred orientations. The black bars show the distribution for strongly orientation-selective pixels (circular variance [CV] < 0.8). The dashed horizontal line indicates expected values for a uniform distribution.

Orientation restriction provides an opportunity to evaluate quantitatively how structured visual experiences influence the functional development of single neurons in a manipulated environment. To guarantee that the animals always experienced a particular orientation, we fabricated goggles with cylindrical lenses (+67 diopter) that allow optical patterns to be transmitted in a severely restricted orientation range (90° ± 12° at 0.5 cycles/degree [cpd], 90° ± 38° at 0.15 cpd)^6^,^7^. These goggles (v-goggles) were chronically mounted on a kitten’s head securely at approximately 3 weeks after birth so that it experienced the vertical orientation exclusively throughout its life (Fig. 1).

To examine the distribution of preferred orientations in the early visual cortex, optical imaging based on intrinsic signals^14^ was conducted at 2 weeks after the v-goggles were attached. As shown previously^6^,^7^, our experimental manipulation induced a rapid and drastic alteration of the orientation maps in which the experienced orientation was predominantly represented in the early visual cortex. Figure 1c shows an example of a cortical orientation map obtained in v-goggled cats. When pixels in area 17 were classified according to their preferred orientation, 69% of the cortical surface was devoted to within ±22.5° of the experienced orientation, spanning only 25% of the orientation domain. Therefore, more than twice the cortical surface was allocated to a limited orientation range in goggle-reared animals than in normal animals^14^. If this analysis was limited to strongly orientation-selective pixels (circular variance < 0.8), this value rose to 85%. This overrepresentation of the exposed orientation is consistent with a previous study^5^, although the effect appears more robust in our case, confirming that the desired restriction of visual experience was achieved. The v-goggles were applied continuously until single-cell electrophysiological recordings were performed when the cats were aged 9 weeks or more.

### Receptive fields are elongated in neurons tuned to the experienced orientation

**Figure 2:**
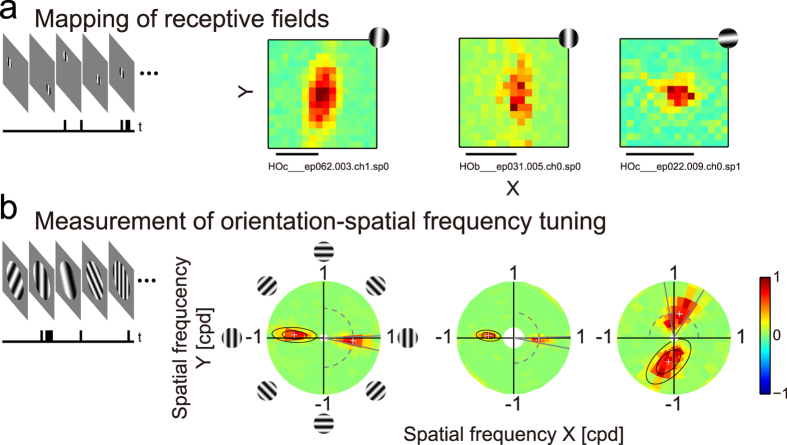
Receptive fields and responses to gratings of different orientations and spatial frequencies for 3 neurons. Data for each cell are shown in separate columns. (**a**) Receptive fields were mapped by presenting a small optimal grating patch at various positions. Stimuli are shown to scale for each neuron in the upper right corner. Scale bars, 5°. (**b**) Two-dimensional tuning to orientation and spatial frequency was examined by presenting gratings of various orientation and spatial frequency combinations. The tuning surface is represented in polar coordinates common to all neurons to simplify comparisons (up to 1 cpd, the perimeter of the circular area). Spatial frequencies of the stimuli were spaced equally on a logarithmic scale, hence the coarser appearance at higher frequencies. The crosshairs denote the peak position of the Gaussian function fitted to the tuning surface (i.e., preferred orientation and spatial frequency), while the inner and outer ellipses indicate the contours at 50% and 10% peak values, respectively. The orientation bandwidth was defined using a 50% criterion, shown as the angle between the two radial grey lines.

To study the effect of orientation-restricted rearing on various tuning properties of individual neurons, single unit activities were recorded using a pair of tungsten microelectrodes inserted into the primary visual cortex of anaesthetised and paralysed cats. Seventeen penetrations were made oblique to the cortical surface in three v-goggled cats. For comparison, the same set of data was also collected from three age-matched normal cats (17 oblique penetrations). Figure 2 shows the responses of representative neurons in v-goggled cats. The top panel (Fig. 2a) shows the overall shape of their receptive fields (i.e., tuning to spatial position) mapped by presenting small grating patches in various locations. Two neurons tuned to the vertical orientation (Fig. 2a, left and middle) exhibited vertically elongated receptive fields, whereas the third neuron tuned to the horizontal orientation (Fig. 2a, right) showed a round receptive field.

**Figure 3:**
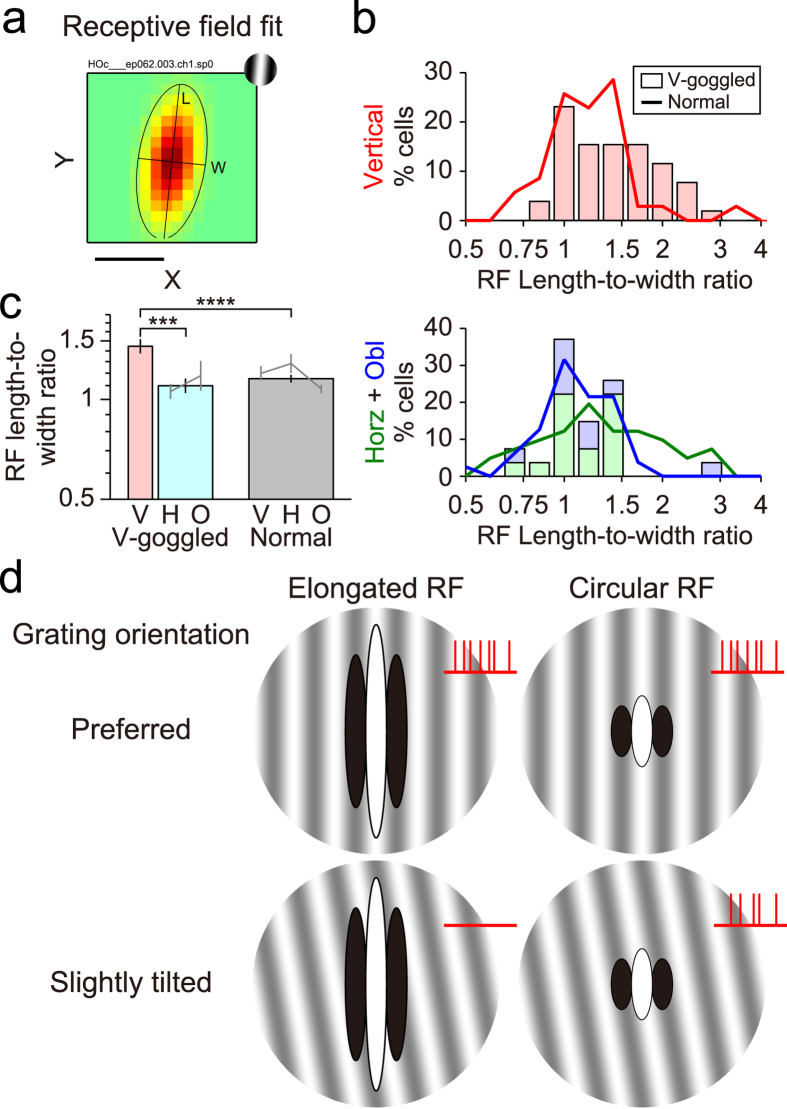
Receptive field elongation of single neurons. (**a**) The spatial extents of receptive fields were determined for each cell by fitting a Gaussian function to the receptive field maps (the same neuron shown in Fig. 2a, left). Their length and width were then quantified relative to the preferred orientations (length, parallel size; width, orthogonal size). (**b**) Distributions of the length-to-width ratio of receptive fields. The histograms show the distributions for v-goggled cats (vertical, n = 51; horizontal, n = 16; oblique, n = 9), whereas the curves show the distributions for normal cats (vertical, n = 35; horizontal, n = 41; oblique, n = 79). The colours indicate 3 groups based on the preferred orientation. As neurons tuned to the horizontal and oblique orientations are scarce and distributed similarly in v-goggled cats, they are plotted in piles. Horz, horizontal; obl, oblique. (**c**) Geometric mean ± standard error of the mean (s.e.m.) of the length-to-width ratio of the receptive fields. The grey bars show the length-to-width ratio for each neuron group. For statistical tests, neurons tuned to the horizontal and oblique orientations were combined into a single group in v-goggled cats (cyan bar), whereas all neurons were combined into a single group in normal cats (grey bar). V, vertical; H, horizontal; O, oblique. ***p < 0.005, ****p < 0.001, the Tukey-Kramer method for multiple comparisons after the Kruskal-Wallis test. (**d**) Receptive fields (RFs) elongated along the preferred orientation predict sharp orientation tuning. In this illustration, 2 types of simple cell receptive fields (elongated [left] vs. circular [right]) are shown on the grating stimulus. Note that when the stimulus is tilted slightly away from the preferred orientation (bottom), the dark parts of the stimulus overlap the ON region (white ellipse) of an elongated receptive field (left) at the edges. Similarly, the bright parts of the stimulus overlap some portions of the OFF regions (black ellipses). Excitation is cancelled in this condition resulting in no spiking response (only this configuration lacks red vertical bars at the top-right corner). This occurs to a much lesser extent for a circular receptive field (right).

Receptive field profiles are closely related to the tuning properties for orientation and spatial frequency^15^,^16^,^17^,^18^,^19^,^20^,^21^ as described briefly in the next subsection. Thus, two parameters were first extracted for each neuron to characterise the receptive field shape by its aspect ratio (Fig. 3a). Namely, length was measured along the direction parallel to the preferred orientation, while width was measured along the direction orthogonal to the preferred orientation. The ratio of these values was then calculated to describe the overall shape of the receptive field. In v-goggled cats, the length-to-width ratio of the receptive fields was 1.45 ± 1.42 on average (geometric mean ± standard deviation [s.d.]) for neurons tuned to the vertical orientation (n = 51), which was significantly higher than that in those tuned to the non-vertical orientations (1.10 ± 1.28, n = 25; p < 0.005, Tukey-Kramer method for multiple comparisons after the Kruskal-Wallis test [see also Statistical tests in the Methods]; hereafter described as the Tukey-Kramer multiple-comparison test for brevity; Fig. 3b,c). Therefore, the receptive fields of neurons tuned to the vertical orientation were elongated vertically by more than 40%, whereas those tuned to the other orientations had nearly circular receptive fields. When defining the groups for comparison, we initially grouped neurons from both v-goggled and normal animals into vertical, oblique, and horizontal, because there is a slight orientation-dependency of image statistics inherent to natural scenes such as the reduced incidence of oblique orientations^22^,^51^. Such groups are retained in the plots of Fig. 3c, but for statistical analysis, neurons tuned to the horizontal and oblique orientations were treated as a single group because they were relatively rare. Accordingly, neurons from age-matched normal cats were also treated as a single group irrespective of their preferred orientation. Compared with the normal control group (1.15 ± 1.38; n = 155), the average length-to-width ratio of receptive fields was still higher for neurons tuned to the vertical orientation in v-goggled cats (p < 0.001, Tukey-Kramer multiple-comparison test), while it was comparable with those tuned to the non-vertical orientations in v-goggled cats (p = 0.64, Tukey-Kramer multiple-comparison test). Therefore, in addition to the vertical-nonvertical difference within the orientation-restricted cortex, the difference persisted in comparison to the normal cortex.

When the length-to-width ratio of receptive fields was plotted against the recorded depth, no relationship was found for neurons that were tuned to the vertical orientation in v-goggled cats (data not shown). In addition, none of the tuning parameters described in the next subsection appeared to have a relationship with cortical depth. Thus, the functional properties of single neurons examined in this study (i.e., receptive field shape, orientation tuning bandwidth, preferred spatial frequency, and spatial frequency bandwidth) were not different across cortical layers (but see also Kreile *et al.*^8^). This analysis was not performed for neurons tuned to the non-vertical orientations in v-goggled cats because the sample size was limited.

### Sharpening of orientation tuning for vertical orientation, and broadening of tuning for non-vertical orientations

Linear systems analysis predicts that a simple-like (or Gabor-like) receptive field elongated along its preferred orientation has sharper tuning than a non-elongated, circular one^15^,^16^,^17^. While this can be characterised quantitatively via Fourier analysis, qualitatively stated, the responses of simple cells are fundamentally determined based on the degree of overlap between the ON regions and the bright parts of stimuli and between the OFF regions and the dark parts of stimuli, i.e., the responses are strongest for perfect overlaps and become weaker for smaller overlaps. Figure 3d demonstrates that elongated receptive fields lose such overlaps when the grating stimulus is tilted slightly away from the preferred orientation (bottom left). In this configuration, the simple cell does not fire spikes. Therefore, elongated receptive fields along the preferred orientation respond to a very limited range of orientation (i.e., sharp orientation tuning). Conversely, circular receptive fields significantly maintain the overlaps (bottom right) and respond to the tilted grating (broad orientation tuning). The same prediction also holds true for complex cells^18^,^19^,^20^,^21^. As described in the previous subsection, in v-goggled cats, the receptive fields were more elongated along the preferred orientation for neurons tuned to the vertical orientation than for those tuned to the non-vertical orientations (Fig. 3). This implies that neurons tuned to the vertical orientation would be more narrowly tuned to their preferred orientation than those tuned to the non-vertical orientations.

To test this, neuronal responses were examined by presenting gratings of various orientation and spatial frequency combinations^23^,^24^. Examples of the measurements are presented in Fig. 2b. In these polar coordinates, stimulus orientation changed along the tangential direction (e.g., the dashed arc). As shown in the same column as in Fig. 2a, these neurons had elongated receptive fields along the preferred orientation. The fact that these neurons were tuned to the vertical orientation can be confirmed by the tangential position of the response peak in Fig. 2b (crosshairs). Remarkably, these two neurons responded to a very narrow range of orientation, which is appreciated by the acute angles made by the two grey lines around the response peak (the grey lines indicate the 50% level of the response peak; orientation bandwidth), demonstrating that these neurons show substantially sharp tuning to orientation. Conversely, a relatively broad range of orientation elicited responses for the neuron that was tuned to the horizontal orientation and had a circular receptive field (Fig. 2b, right). These relationships between receptive field shape and orientation tuning bandwidth are consistent with the above prediction.

**Figure 4:**
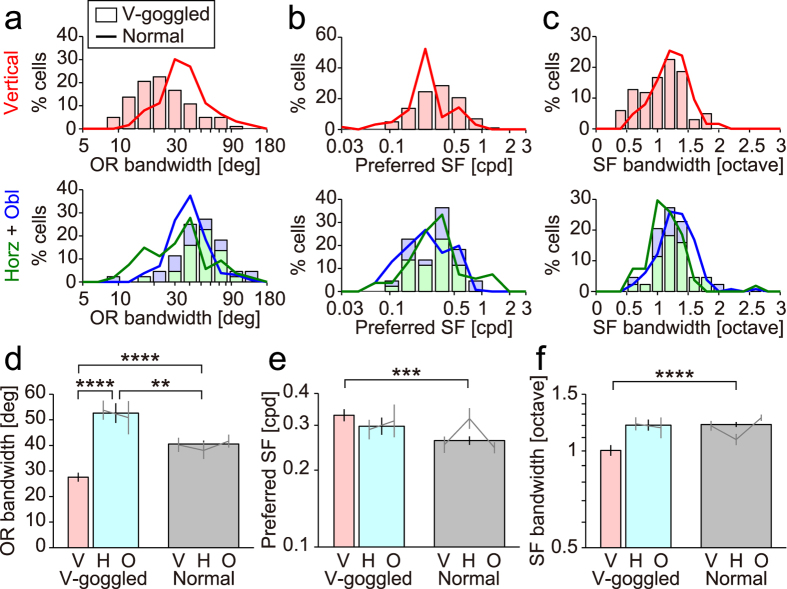
Orientation and spatial frequency tuning of single neurons. The orientation and spatial frequency tuning parameters of individual neurons were obtained by fitting a Gaussian function to the response surfaces of each cell (Fig. 2b). In **a**–**c**, the histograms show the distributions for v-goggled cats (vertical, n = 102; horizontal, n = 27; oblique, n = 17), whereas the curves show the distributions for normal cats (vertical, n = 63; horizontal, n = 54; oblique, n = 131). The number of cells was reduced for spatial frequency bandwidth to define the high cutoff frequency in both v-goggled (vertical, n = 98; horizontal, n = 26; oblique, n = 13) and normal cats (vertical, n = 57; horizontal, n = 51; oblique, n = 123). The colours indicate 3 groups based on the preferred orientation. The error bars in d–f indicate s.e.m. Horz, horizontal; obl, oblique. V, vertical; H, horizontal; O, oblique. **p < 0.01, ***p < 0.005, ****p < 0.001, Tukey-Kramer method for multiple comparisons after the Kruskal-Wallis test.

Figure 4 summarises the orientation and spatial frequency tuning parameters of individual neurons in v-goggled and normal cats. In v-goggled cats, neurons tuned to the vertical orientation had a significantly narrower orientation tuning bandwidth (full width at half height) (27.6° ± 17.7° [mean ± s.d.], n = 102; Fig. 4a,d) than those tuned to the non-vertical orientations (52.6° ± 25.5°, n = 44; p < 0.001, Tukey-Kramer multiple-comparison test). More notably, orientation bandwidth for neurons tuned to the vertical orientation in v-goggled cats was narrower than that for cells in normal cats (40.5° ± 20.9°, n = 249; p < 0.001, Tukey-Kramer multiple-comparison test). Conversely, neurons tuned to the non-vertical orientation in v-goggled cats had a broader orientation bandwidth than that for cells in normal cats (p < 0.01, Tukey-Kramer multiple-comparison test).

In v-goggled cats, neurons tuned to the vertical orientation generally preferred a similar spatial frequency (vertical, 0.33 ± 1.74 cpd [geometric mean ± s.d.], n = 102; Fig. 4b,e) compared to those tuned to the non-vertical orientations (0.30 ± 1.67 cpd, n = 44; p = 0.66, Tukey-Kramer multiple-comparison test). However, the former value was significantly higher than that of cells in normal cats (0.26 ± 1.84, n = 249; p < 0.005, Tukey-Kramer multiple-comparison test), while the latter was comparable (p = 0.37, Tukey-Kramer multiple-comparison test). As for spatial frequency bandwidth, in v-goggled cats, neurons tuned to the vertical orientation appeared to show smaller values (1.00 ± 1.45 octave [geometric mean ± s.d.], n = 98; Fig. 4c,f) than those tuned to the non-vertical orientations (1.20 ± 1.28 octave, n = 39), but the difference did not reach statistical significance (p = 0.06, Tukey-Kramer multiple-comparison test). The former bandwidth was significantly narrower than that of cells in normal cats (1.21 ± 1.31 octave, n = 231; p < 0.001, Tukey-Kramer multiple-comparison test), whereas the latter was comparable (p = 0.90, Tukey-Kramer multiple-comparison test).

**Figure 5:**
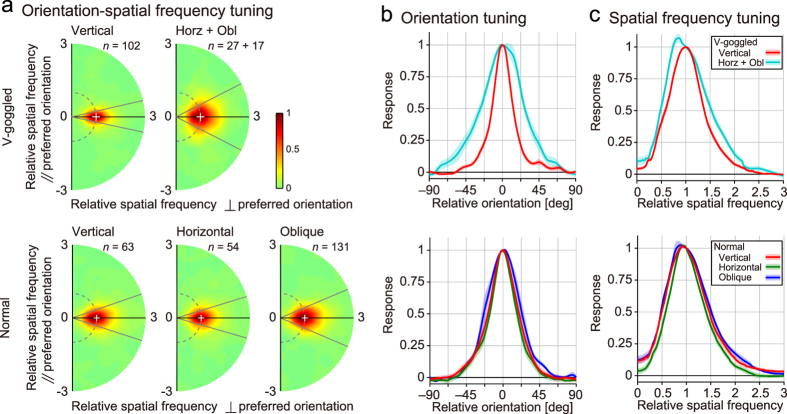
Tuning curves averaged across a population of neurons in area 17. (**a**) Orientation-spatial frequency tunings of all neurons were averaged after rotating and scaling individual maps such that the preferred orientation and spatial frequency became 90° and 1, respectively (white crosshairs). Tuning for cells tuned to the vertical (top left) and non-vertical (horizontal + oblique) orientations (top middle) from v-goggled cats, and for those tuned to the vertical (bottom left), horizontal (bottom middle), and oblique (bottom right) orientations from normal cats. The orientation bandwidth is shown as the angle between the two radial grey lines, as in Fig. 2b. Horz, horizontal; obl, oblique. (**b**) Orientation tuning curves obtained as cross-sections along the dashed arcs through the peaks in a. The translucent bands indicate s.e.m. Top, v-goggled cats. Bottom, normal cats. Red, cells tuned to the vertical orientation; cyan, non-vertical orientation; green, horizontal orientation; and blue, oblique orientation. (**c**) Spatial frequency tuning curves obtained as cross-sections along the horizontal black lines through the peaks in a.

To visualise orientation and spatial frequency tuning for our sample of neurons, their tuning surfaces were averaged after aligning their preferred orientation and normalizing the spatial frequency scale (Fig. 5a). For this analysis, the tuning surface of each neuron was first rotated and scaled such that the preferred orientation and spatial frequency became 90° and 1, respectively. Then, these tuning surfaces were averaged across neurons within cell groups to summarise them. Tuning curves to orientation and spatial frequency were finally obtained by taking the cross-sections of the two-dimensional tuning profiles in those tuning surfaces through the peak (along the dashed arc for orientation tuning in Fig. 2b; along the radial direction through the peak for spatial frequency tuning). Although the effects were reduced to some extent due to imperfect alignment of the tuning surfaces across neurons, orientation (Fig. 5b) and spatial frequency tuning curves (Fig. 5c) obtained for different cell groups illustrated the above numerical description in an intuitive manner.

### Population response is not minimal for orientation orthogonal to the experienced one

**Figure 6:**
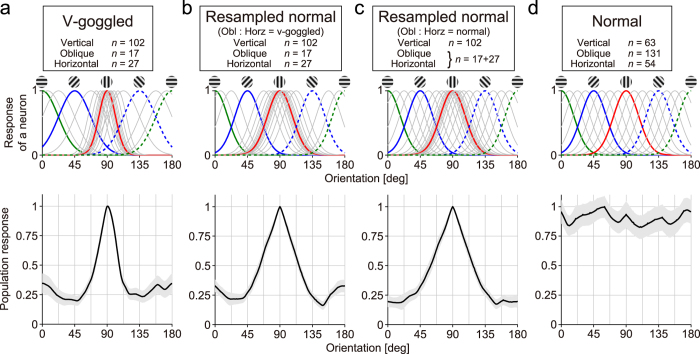
Orientation representation by a population of neurons in area 17. Top, Schematic illustration of orientation tuning curves of single neurons. The density of overlaid curves indicates the approximate relative number of cells (curves were removed for clarity when needed). Five curves are highlighted in colours for visual purposes. Bottom, Population orientation tunings were obtained as follows. Two-dimensional tuning surfaces were averaged across neurons. By integrating the plot radially (thereby removing the spatial frequency dimension), population tuning to orientation was obtained. The grey bands indicate the s.e.m. of the bootstrap distributions. (**a**) Tuning for v-goggled cats. (**b**) Simulated tuning. Data from normal cats were resampled so that the number of cells in each of the 3 orientation groups matched that from v-goggled cats. (**c**) Simulated tuning. Data from normal cats were resampled so that the number of cells tuned to the vertical and those tuned to the non-vertical orientations (i.e., horizontal + oblique as a single group) matched that from v-goggled cats. In this curve, the ratio of the number of cells for the non-vertical orientations (horizontal vs. oblique) was identical to that in normal cats. (**d**) Tuning for normal cats.

We next examined how a population of area 17 neurons in v-goggled cats represents orientation. Figure 6a illustrates the likely answer to this question. As shown above, cells were sharply tuned to orientation if they preferred the vertical orientation, whereas they were broadly tuned otherwise. In agreement with previous studies^2^,^3^,^4^,^5^,^6^,^7^,^8^,^9^ and our optical imaging data, a majority of neurons (102 of 147 cells) were tuned to the vertical orientation. Interestingly, the effects were not equal for the non-experienced orientations; although the oblique orientation was closer to the experienced orientation than the horizontal orientation, there were fewer neurons tuned to the oblique orientation than the horizontal orientation (17 vs. 27 cells, respectively). This difference could not be due to the residual oblique effect found in normal adult animals^25^,^26^,^27^. First, these numbers for v-goggled cats were unlikely to be observed by chance when the distribution of cells in normal adult cats^26^ was assumed (p < 0.005, binomial test). Second, the corresponding numbers in age-matched control cats (131 vs. 54 cells, respectively) did not support the oblique effect. As a consequence, when an orientation tuning curve for a population of neurons was obtained by summing individual orientation tuning curves for recorded neurons (Fig. 6, bottom), the population tuning curve exhibited significantly lower responses to the oblique orientation (45° and 135°) than to the horizontal orientation (0°) (p < 0.001, resampling; Fig. 6a). This was not explained by the oblique effect^25^,^26^,^27^ in normal cats either (Fig. 6d), because such local troughs did not emerge around the oblique orientation in the tuning curve when neurons from normal cats were resampled so that cells tuned to the oblique and horizontal orientations were selected at the same probability as in normal cats (Fig. 6c; compare with Fig. 6b). Furthermore, population orientation tuning was significantly narrower in the actual v-goggled cats than in the resampled data (Fig. 6a vs. b; p < 0.001, resampling), reflecting the sharpened orientation tuning of single neurons tuned to the vertical orientation in v-goggled cats.

## Discussion

This study demonstrates that the functional properties of single neurons in the primary sensory cortex can be tailored to a particular environment experienced by animals in early life. We showed that the orientation selectivity of area 17 neurons was modulated based on the experience of kittens reared with chronically mounted goggles. More specifically, orientation bandwidth became narrower for neurons tuned to the experienced orientation, whereas the opposite was true for those tuned to the non-experienced orientations. This means that neuronal selectivity reflects the frequency of stimuli in a sensory environment (sharp tuning for abundant stimuli and broad tuning for rare stimuli). Orientation selectivity appeared to be sharpened, at least partly, by the elongation of receptive fields for neurons tuned to the experienced orientation. In addition to the well-appreciated increase in the proportion of neurons tuned to the experienced orientation^2^,^3^,^4^,^5^,^6^,^7^,^8^,^9^, we found that the primary visual cortex in orientation-restricted animals did not have neurons uniformly for the non-experienced orientations; neurons tuned to orientations close to the experienced one are most likely to shift their orientation preference toward the experienced one^5^,^8^ because their orientation bandwidth can cover the experienced orientation.

Although other possibilities cannot be ruled out, the above results are highly consistent with a theoretical prediction^13^ in which the receptive fields of single neurons are developed through sparse coding^28^,^29^,^30^ for orientation-restricted natural images. Hsu and Dayan^13^ accurately predicted that orientation tuning becomes sharper and broader for neurons tuned to vertical and non-vertical orientations in v-goggled cats, respectively. The sharpened orientation tuning of cells tuned to the vertical orientation can be interpreted as a strategy to sparsify an orientation representation among these cells because sharper tuning reduces the range of orientation for individual cell signalling. In addition, among the decreased number of cells for the non-vertical orientations, the greater loss of neurons tuned to the oblique orientation would contribute to a sparser code by reducing the number of cells that are activated weakly by the vertical orientation, which is represented by a separate set of neurons. However, there was one prediction that we did not find in our results; there was no significant difference in the distribution of preferred spatial frequencies between neurons tuned to the vertical orientation and those tuned to other orientations in v-goggled cats, although the former neurons tended to prefer higher spatial frequencies with narrower bandwidths than neurons in normal cats.

Here, we demonstrate that the primary visual cortex adapts to an orientation-restricted environment by sharpening the orientation tuning of the majority of single neurons, while allocating other cells to non-experienced stimuli in an economical manner through sparse coding^13^,^28^,^29^,^30^. While biological evidence for sparse coding has been shown in animals with normal sensory^16^,^17^,^31^,^32^,^33^ and motor experiences^34^, this report shows it occurs even in an orientation-restricted environment. Therefore, we suggest sparse coding is likely to be a general principle used in early brain areas to represent information efficiently even when their statistical structure does not occur naturally.

This is not the first investigation to examine the orientation bandwidth of single neurons in orientation-restricted animals. Sengpiel *et al.*^5^ reported that orientation restriction generates neurons in area 17 with the same orientation bandwidth independent of their preferred orientation. The apparent contradiction between the findings of Sengpiel *et al.*^5^ and our results might be caused by a difference in the degree of orientation restriction. Orientation was restricted in a modest environment in the previous investigation^5^; the animals spent 3–5 h per day in cylinders painted with stripes of a single orientation and the rest of the time in the dark. In contrast, our animals were always under orientation restriction with chronically mounted goggles when they were awake. As a result, a more modest change was observed in the orientation maps measured with optimal imaging in the former study (approximately 35% of the cortical surface was allocated to the experienced orientation in Sengpiel *et al.*^5^; 69% in our study). In fact, sparse coding predicts quantitatively different effects of orientation restriction based on the proportion of orientation-restricted images to natural images used during learning^13^. Furthermore, this difference in the orientation representation of cortical maps actually explains the inconsistent orientation bandwidth of single neurons because neurons tend to have sharp orientation tuning when they are surrounded by neighbours that share the same orientation preference^35^,^36^.

The majority of neurons in early sensory areas develop preferences to the exposed stimulus when young animals are reared in an environment dominated by a particular sensory stimulus^2^,^3^,^4^,^5^,^6^,^7^,^8^,^9^,^10^,^11^,^12^. As replicated in this study, the primary visual cortex has many neurons that prefer the vertical orientation in cats whose visual experience is restricted to the vertical orientation (vertical stripe and v-goggle rearing) in youth^2^,^3^,^4^,^5^,^6^,^7^,^8^,^9^. Such sensory coding can be considered as structural specialisation for representing the available stimuli in an environment by allocating a minimum number of neurons to rare stimuli. Conversely, continuous exposure to a particular stimulus for a short period of time (e.g., seconds to hours) diminishes response sensitivity or gain to the exposed stimulus in the brain^37^,^38^,^39^,^40^. By measuring the responses of a population of neurons, a recent study reported that this mechanism equalises the time-averaged responses across the neurons, adjusting the response level of each neuron to match the scarcity of stimuli in an environment^40^. Therefore, this mechanism has an opposing function to long-term changes, i.e., sensitisation to rare stimuli. When young animals are exposed to a particular stimulus continuously, the early sensory areas experience two different phases of adaptation: an initial decrement in the sensitivity of single neurons to abundant stimuli and eventual increment in the number of neurons responding to them. Although these two phases have opposite effects, physiological investigations have found evidence for both forms of plastic change^2^,^3^,^4^,^5^,^6^,^7^,^8^,^9^,^37^,^38^,^39^,^40^,^41^,^42^,^43^. In addition, a third form of adaptation has been discovered (with a time course between the above two stages) by psychophysical studies in which perceptual effects were examined in human subjects using orientation restriction experiments^44^,^45^,^46^. When the visual experience was restricted to orientations other than the vertical orientation using head-mounted displays for a period of days, humans show biphasic adaptation effects in perceptual tasks for contrast and orientation judgment^46^. Haak *et al.*^46^ reported that such a transition requires approximately 1 day to occur. Orientation restriction for 1 day presumably does not cause a structural change in the distribution of orientation preferences in the population of neurons in the primary visual cortex as reported in many studies^2^,^3^,^4^,^5^,^6^,^7^,^8^,^9^; otherwise, it is very difficult to recover from such a drastic alteration. Thus, sensitivity can be diminished both with and without the cortical dominance of neurons responding to the exposed stimuli. At least three different stages may be involved in this process, eventually leading to the long-term induction of orientation restriction^2^,^3^,^4^,^5^,^6^,^7^,^8^,^9^. The molecular and cellular mechanisms for each adaptive stage and those triggering transitions across them have yet to be elucidated.

## Methods

All animal care and experimental guidelines were in accordance with those established by the US National Institutes of Health and were approved by the Institutional Animal Research Committee at RIKEN (intrinsic optical imaging) and by the Osaka University Animal Care and Use Committee (electrophysiological recordings). The experiments were conducted on anaesthetised and paralysed cats.

### Orientation restriction with v-goggles

For orientation restriction, we fabricated goggles with cylindrical lenses (+67 diopters) so that optical patterns could be transmitted in a severely limited orientation range (90° ± 12° at 0.5 cpd, 90° ± 38° at 0.15 cpd)^6^,^7^. These goggles were mounted on a kitten’s skull securely under anaesthesia at approximately 3 weeks after birth as described previously^6^,^7^. The animals experienced the same orientation exclusively throughout their lives. Exposure to unrestricted orientations was minimised (<2 min) for the daily check and care of the goggles.

### Optical imaging based on intrinsic signals and data analyses

The procedures for animal preparation and maintenance, surgery, and experiment setup for optical imaging based on intrinsic signals have been described in detail elsewhere^6^,^7^. Only a brief account is provided here. Cortical images were captured in area 17/18 under the illumination of 700-nm light, while full-field square-wave gratings were drifted in 12 directions monocularly (0.15 and 0.5 cpd). An orientation map was calculated using a conventional vector sum method^5^,^14^ for responses averaged across eyes and spatial frequencies. Circular variance^47^ was also calculated for these responses to quantify orientation selectivity. As neurons in area 17 tend to prefer a higher spatial frequency than those in area 18^48^, pixels included in area 17 were determined based on higher responses to 0.5 cpd gratings than to 0.15 cpd gratings^49^.

### Electrophysiology and data analyses

The procedures for animal preparation and maintenance, surgery, single-unit recording, and experiment setup have been described in detail elsewhere^21^,^50^. Only a brief account is provided here, with an emphasis on those aspects of the methodology most relevant to the present study. Single units were recorded in the oblique penetrations of tungsten microelectrodes in area 17 of 3 v-goggled (at 63, 84, and 99 days after birth) and 3 normal cats (at 63, 77, and 98 days after birth). Microelectrodes penetrated the grey matter obliquely (~20°) to avoid a bias arising from recording continuously in the same cortical (i.e., orientation) columns. Seventeen penetrations were made in both v-goggled and normal cats.

To map receptive fields, a small optimal grating was presented at various positions in rapid succession (typically 76/3 Hz). The diameter of these gratings was equal to 3 sampling points and was customised for each neuron to maximise both the responses and spatial resolution. The obtained maps were fitted with a 2D Gaussian function to determine the spatial extent of the receptive fields at the 10% peak value. Length was measured along the directions of the preferred orientations determined below, whereas width was measured along their orthogonal directions.

To examine orientation and spatial frequency tunings, sinusoidal gratings of various orientation and spatial frequency combinations were presented in rapid succession (typically 76/3 Hz)^23^,^24^. Grating size was adjusted for each neuron to cover the receptive fields entirely (typically, 1.5–3 times larger than the receptive fields). Orientations were typically sampled at 10° steps. If necessary, however, the sampling interval was reduced around the preferred orientation for neurons that showed very sharp tuning. Spatial frequencies were sampled at equal logarithmic steps over a range customised for each cell. The tuning surfaces were fitted with a 2D Gaussian function to extract the centre position (preferred parameters) and the spread of responses (bandwidths). Specific analyses are described at the relevant places in the Results.

### Statistical tests

To compare stimulus selectivity across neuronal groups, the Tukey-Kramer method was used to correct for multiple comparisons after the Kruskal-Wallis test. In these tests, medians were compared across 3 neuronal groups: neurons tuned to the vertical orientation in v-goggled cats, neurons tuned to non-vertical orientations in v-goggled cats, and all neurons in normal cats.

## Additional Information

**How to cite this article**: Sasaki, K. S. *et al.* Supranormal orientation selectivity of visual neurons in orientation-restricted animals. *Sci. Rep.* **5**, 16712; doi: 10.1038/srep16712 (2015).
